# Advancing early gastric cancer detection

**DOI:** 10.1002/2211-5463.13217

**Published:** 2021-07-04

**Authors:** Sarah Gillen

**Affiliations:** ^1^ The Beatson Institute for Cancer Research Glasgow UK

**Keywords:** biomarker, gastric cancer, miRNA, miR‐194, prognosis, the cancer genome atlas

## Abstract

Important factors in combating cancer are early detection and accurate assessment of the best course of treatment. In a study published in this issue, Wang *et al*. identify possible miRNA biomarkers for improved determination of gastric cancer stage and prognosis. In particular, they show increased miR‐194 levels are a predictor of more favourable gastric cancer prognosis, at least in part due to miR‐194 downregulating production of a key protein for cancer development: CCND1.

## Graphical abstract

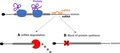

**Abbreviations**
TCGA: The Cancer Genome Atlas
mRNA: messenger RNA

Gastric (stomach) cancer is a commonly diagnosed form of cancer and is third in terms of cancer‐related deaths each year worldwide [1]. A major contributor to the high mortality rate of gastric cancer is that it is often diagnosed at a late stage. Improved detection of gastric cancer could help with determining the best course of treatment and allow for more accurate prediction of the outcome for the patient. Biomarkers are measurable aspects of biology that can be associated with a factor of interest; in the context of cancer, this factor may be diagnosis, survival outcome or response to treatment. MicroRNAs (miRNAs) are short, relatively stable pieces of RNA that can be detected in patient samples, and are becoming popular biomarkers because they are diverse, widely present and change with health and disease [2].

The Cancer Genome Atlas (TCGA) programme [3] is a global effort to collate data from a wide range of cancer types and is a resource accessible to researchers throughout the world. In a study published in this issue of *FEBS Open Bio* [4], Wang *et al*. utilised some of these data from 391 gastric cancer patients to identify miRNAs that could serve as potential biomarkers for this cancer type. High confidence hits were obtained for miRNAs associated with both gastric cancer survival and several cancer stage indicators. MiRNAs can be characterised as oncogenic, meaning they promote cancer development, or tumour suppressive, meaning they prevent cancer progression [5]. In this study, Wang *et* *al*. highlight three high confidence oncogenic miRNAs (miR‐100, miR‐125b and miR‐199a) and one tumour‐suppressive miRNA (miR‐194).

Wang *et al*. focussed in on miR‐194. They observed that the greater the amount of miR‐194 present, the more favourable the gastric cancer prognosis. They also showed a reduction in miR‐194 levels as the tumour stage progresses, suggesting miR‐194 acts a tumour suppressor [4]. This led the researchers to ask *how* miR‐194 leads to favourable prognosis of gastric cancer.

MiRNAs generally act to reduce protein production from their target messenger RNA (mRNA) molecules. The absence of a specific protein can dramatically alter the behaviour of a cancer cell. There are two main ways in which miRNAs function: either miRNAs can target the mRNA molecule for destruction which removes the instructions for the production of a specific protein from the cell, or they can prevent the synthesis of the protein encoded in the mRNA (Fig. 1).

**Fig. 1:**
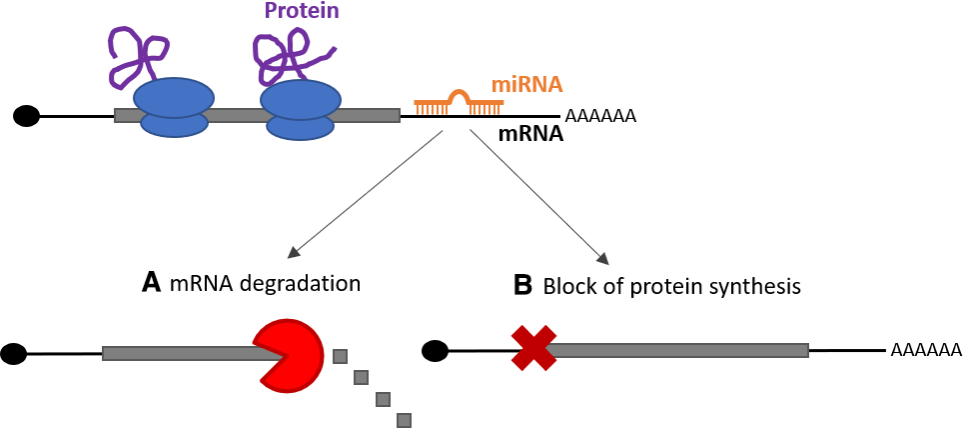
Mechanisms by which miRNAs regulate mRNAs. MiRNAs primarily bind towards the end of the mRNA and prevent protein production by either (A) recruiting factors to degrade the mRNA so this information to produce the protein is no longer present in the cell or (B) bringing factors to the mRNA that block the synthesis of the protein encoded in the mRNA.

Wang *et al*. used a method called RNA sequencing to identify mRNA molecules expressed in gastric cancer cells which are controlled by miR‐194. They uncovered that miR‐194 is likely associated with better prognosis due to its regulation of a gene named *CCND1* [4]. The encoded protein CCND1 plays an important role in cell division, which in turn is a key component of cancer development. Therefore, it is likely that the reduced cell growth and suppressed cancer progression observed in the presence of miR‐194 are a result of downregulated CCND1 levels [4], although additional targets of miR‐194 identified through RNA sequencing have not been ruled out. The researchers demonstrated that miR‐194 controls CCND1 levels in two different sets of gastric cancer cells, strengthening their findings. Nevertheless, this RNA sequencing method can only detect mRNAs targeted for destruction by miR‐194 (as in Fig. 1A), and not those for which miR‐194 prevents protein synthesis without degrading the mRNA (as in Fig. 1B). This aspect could be investigated further in the future work to increase our understanding of the function of miR‐194 in gastric cancer and possibly identify additional important targets.

Another recent study has tested the potential for using a set of circulating miRNAs as biomarkers for the detection of gastric cancer in patient serum samples [6]. If the miRNAs identified in the study by Wang *et al*. are also detectable in the population of circulating miRNAs, they may be useful additions to improve the panel of miRNAs used for gastric cancer detection, stage identification and prognosis assessment in the future.

## Conflict of interest

The authors declare no conflict of interest.

## Author contribution

SG wrote the article and prepared the figure.
